# Resting-state brain functional alterations and their genetic mechanisms in drug-naive first-episode psychosis

**DOI:** 10.1038/s41537-023-00338-z

**Published:** 2023-02-25

**Authors:** Qian Li, Xiaotao Xu, Yinfeng Qian, Huanhuan Cai, Wenming Zhao, Jiajia Zhu, Yongqiang Yu

**Affiliations:** 1 Department of Radiology, Chaohu Hospital of Anhui Medical University, 238000 Hefei, China; 2 Department of Radiology, The First Affiliated Hospital of Anhui Medical University, 230022 Hefei, China; 3 Research Center of Clinical Medical Imaging, Anhui Province, 230032 Hefei, China; 4 Anhui Provincial Institute of Translational Medicine, 230032 Hefei, China

**Keywords:** Schizophrenia, Biomarkers

## Abstract

Extensive research has established the presence of resting-state brain functional damage in psychosis. However, the genetic mechanisms of such disease phenotype are yet to be unveiled. We investigated resting-state brain functional alterations in patients with drug-naive first-episode psychosis (DFP) by performing a neuroimaging meta-analysis of 8 original studies comprising 500 patients and 469 controls. Combined with the Allen Human Brain Atlas, we further conducted transcriptome-neuroimaging spatial correlations to identify genes whose expression levels were linked to brain functional alterations in DFP, followed by a range of gene functional characteristic analyses. Meta-analysis revealed a mixture of increased and decreased brain function in widespread areas including the default-mode, visual, motor, striatal, and cerebellar systems in DFP. Moreover, these brain functional alterations were spatially associated with the expression of 1662 genes, which were enriched for molecular functions, cellular components, and biological processes of the cerebral cortex, as well as psychiatric disorders including schizophrenia. Specific expression analyses demonstrated that these genes were specifically expressed in the brain tissue, in cortical neurons and immune cells, and during nearly all developmental periods. Concurrently, the genes could construct a protein-protein interaction network supported by hub genes and were linked to multiple behavioral domains including emotion, attention, perception, and motor. Our findings provide empirical evidence for the notion that brain functional damage in DFP involves a complex interaction of polygenes with various functional characteristics.

## Introduction

Schizophrenia, a severe and chronic psychiatric condition with a lifetime prevalence of about 1%^1^, is characterized by hallucinations, delusions and cognitive deficits. It is generally accepted that many of the clinical manifestations thought to be characteristic of psychosis may be attributable to abnormal brain function^2,3^. Given the presence of spontaneous neural activity along with intrinsic metabolic and perfusion patterns in the brain, considerable effort in the past decade has been dedicated to examine resting-state brain function in health and disease^4–7^. In this context, resting-state brain function has been frequently measured by amplitude of low-frequency fluctuations (ALFF) and regional homogeneity (ReHo) derived from functional magnetic resonance imaging (fMRI)^8–10^ as well as by cerebral blood flow (CBF) derived from arterial spin labeling (ASL) or positron emission tomography (PET)^11,12^. Use of these resting-state brain functional measures facilitates generalization of findings in the domain of psychiatry, given that collection of these data is now commonplace in semi-standardized ways and is less prone to the confounding effects of variation in task compliance. By leveraging these approaches, extensive research has established the presence of resting-state functional alterations in psychosis^7,13–18^, but inconsistency in the location and nature of effects makes it difficult to unify this research. This is largely due to the fact that most prior studies have focused on antipsychotic-medicated and chronic psychosis patients, which may introduce various confounds of antipsychotic medication and illness duration.

Benefiting from factoring out the confounds of antipsychotics and illness chronicity, investigations of resting-state brain functional alterations in patients with drug-naive first-episode psychosis (DFP) have attracted intense interest from researchers^19–26^. However, the reliability of these previous studies has been challenged by the concerns of small sample sizes, clinical heterogeneity, and flawed correction for multiple comparisons, which jointly work to inflate false positive rates. Despite the limitations, these studies set the stage for neuroimaging meta-analysis, a useful method that is capable of enlarging sample size and increasing statistical power by a more in-depth synthesis of findings in the literature. With the use of neuroimaging meta-analysis, investigators have enjoyed significant success in unraveling reliable and reproducible brain structural and functional changes in DFP^27–30^.

Psychosis is a highly heritable disorder with heritability estimated at up to 79%^31,32^. Although a large-scale genome-wide association study (GWAS) has identified many risk genetic loci in association with schizophrenia^33^, relatively little is known about the exact genetic mechanisms underlying certain disease phenotypes, such as brain functional damage. The integration of brain-wide gene expression data such as the Allen Human Brain Atlas (AHBA)^34^ and brain imaging data has given rise to the nascent field of neuroimaging transcriptomics^35–38^. In this framework, spatial correlations between brain transcriptome and disease neuroimaging phenotypes are a topic of active investigation, which may aid in the identification of genes whose expression profiles are associated with brain structural and functional change patterns in psychiatric conditions^39–47^. By means of this powerful method, several earlier studies have discovered several sets of genes in relation to abnormal brain structural properties in psychosis, such as structural covariance network deficiency^39^, white matter dysconnectivity^40^, morphometric similarity abnormality^41^, and gray matter volume changes^42^. Nevertheless, the genetic mechanisms of brain functional alterations in DFP are yet to be unveiled.

**Fig. 1:**
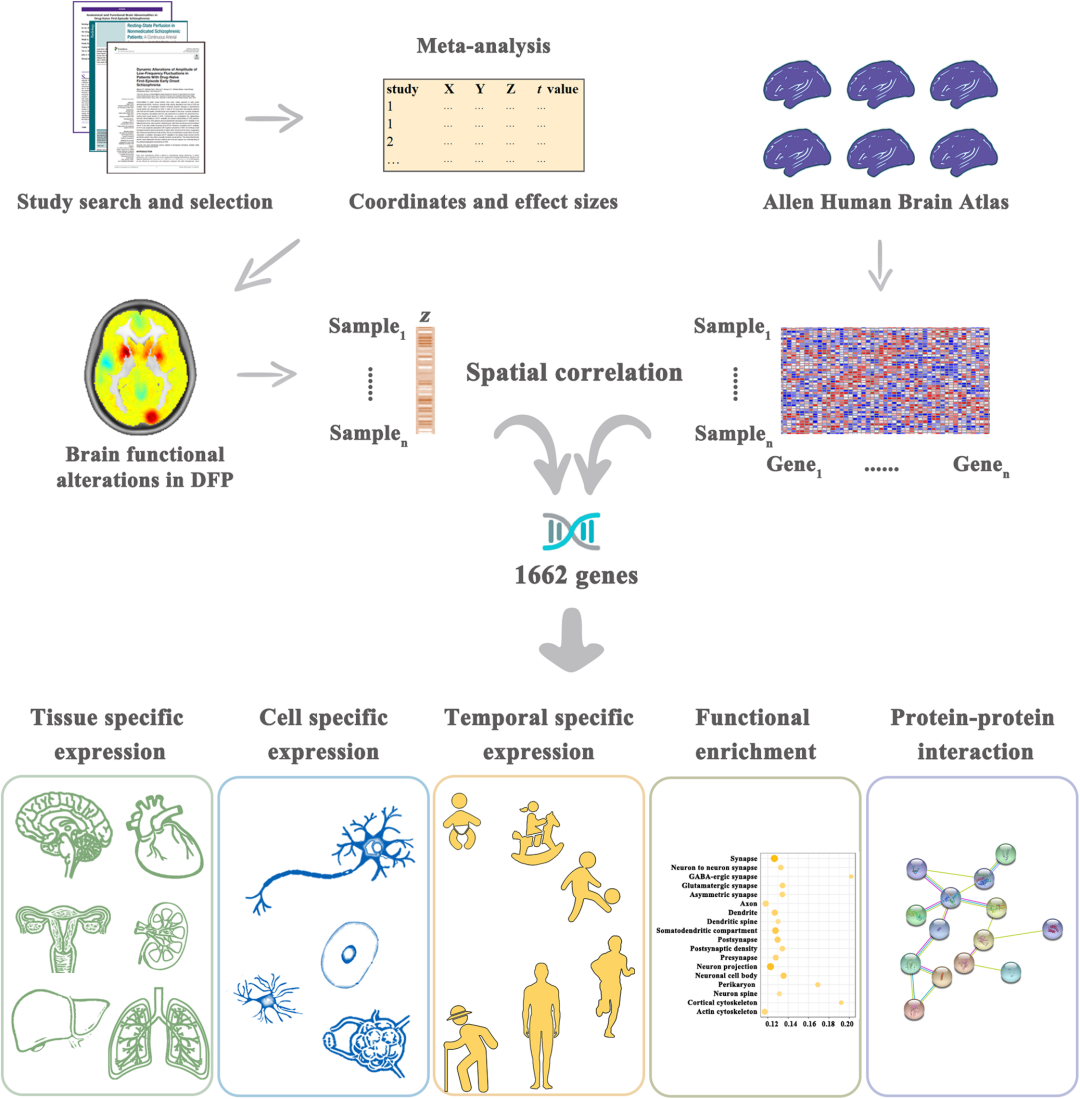
A schematic summary of the analysis pipeline. DFP patients with drug-naive first-episode psychosis.

To this end, a comprehensive neuroimaging meta-analysis of ALFF, ReHo, and CBF was initially performed to investigate resting-state brain functional alterations in DFP. In conjunction with the AHBA, transcription-neuroimaging spatial correlation analyses were then conducted to detect genes whose expression levels were linked to brain functional alterations in DFP. Finally, we carried out a series of post hoc analyses (i.e., specific expression, functional enrichment, and protein-protein interaction analyses) for the identified genes to examine their functional characteristics. A schematic summary of the analysis pipeline is shown in Fig. 1.

## Methods

### Literature search and selection

The literature search strategy was conducted in accordance with the Preferred Reporting Items for Systematic Reviews and Meta-analyses (PRISMA) guidelines^48^. A systematic search was performed independently by two authors (Q.L. and X.X.) using the PubMed and Web of Science databases to identify studies examining differences in resting-state brain function between DFP and healthy controls (HC), published before February 2021. Considering that brain functional measures involved ReHo, ALFF, and CBF, we used various combinations of the following keywords during the search: “schizophr*”, “psychosis”, “psychotic disorders”, “first episode”, “first episodes”, “1^ST^-EPISODE”, “FEP”, “FES”, “free”, “naive”, “never medicated”, “ReHo”, “regional homogeneity”, “local connectivity”, “local functional connectivity”, “ALFF”, “fALFF”, “LFF”, “LFO”, “low-frequency fluctuation”, “low-frequency fluctuations”, “amplitude of low-frequency fluctuation”, “amplitude of low-frequency fluctuations”, “amplitude of low-frequency oscillation”, “amplitude of low-frequency oscillations”, “CBF”, “brain blood flow”, “cerebral blood flow”, “rCBF”, and “rest*”. We also screened the reference lists of the identified studies, review articles, and meta-analyses to search for additional qualified studies.

**Fig. 2:**
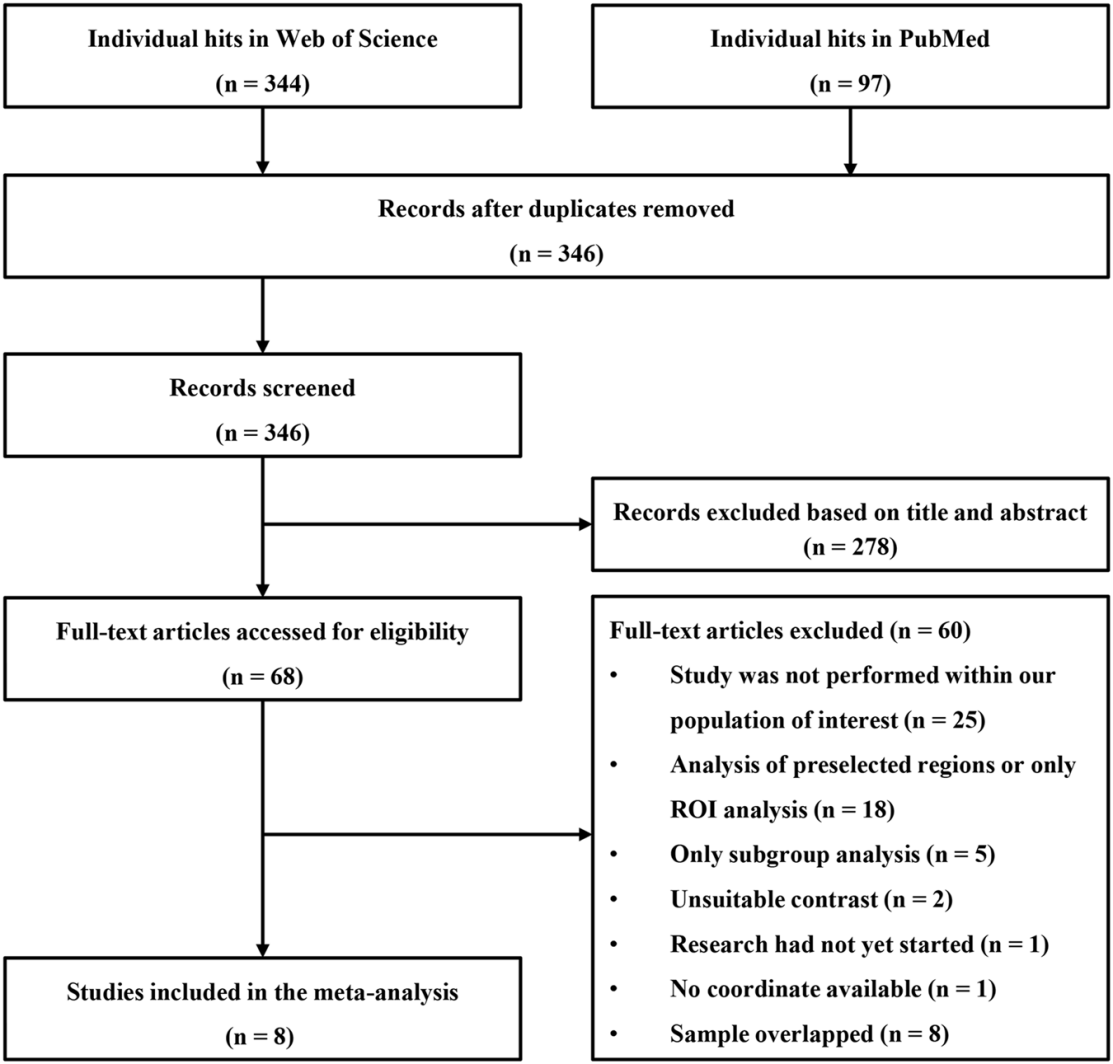
Flowchart of the study selection process. ROI region of interest.

Studies were included if they met the following criteria: (1) studies examined resting-state brain functional differences between DFP and HC in a voxel-wise manner; (2) group comparisons were conducted at the whole-brain level; (3) statistical results were reported in a standard anatomical reference space (Montreal Neurological Institute [MNI] or Talairach space). In cases where coordinates were not reported in published papers, corresponding authors were contacted for details. Studies were excluded based on the following criteria: (1) voxel-wise comparisons were carried out within regions of interest; (2) studies involved animal experiments; (3) data could not be obtained from published papers or after contacting the authors; (4) participants were single genders. Notably, we also excluded studies whose data overlapped with those of other studies (e.g., from the same institution or authors), and the studies with the most results or larger effect sizes were included in our meta-analysis. A flowchart of the detailed study selection process is shown in Fig. 2. This meta-analysis was registered on PROSPERO (https://www.crd.york.ac.uk/PROSPERO/, NO: CRD42021252171).

### Neuroimaging meta-analysis

Voxel-wise meta-analysis of brain functional alterations in DFP was conducted using Seed-based *d* Mapping with Permutation of Subject Images (SDM-PSI) software package (version 6.21 for Windows) in a standard process (https://www.sdmproject.com). In contrast to the traditional activation likelihood estimation (ALE) method, SDM-PSI enables investigators to combine both peak coordinates and statistical parametric maps and uses standard effect size and variance-based meta-analytic calculations. The detailed analysis steps have been described in the SDM-PSI tutorial and previous publications^49,50^. First, we extracted peak coordinates and corresponding effect sizes (e.g., *t*-values) of clusters showing statistically significant differences in brain function between DFP and HC from each study. Coordinates in different standard anatomical reference spaces were converted from Talairach to MNI coordinates by using icbm2tal tools^51^ and *z*- or *P-*values were converted to *t*-values by using SDM online conversion utilities (https://www.sdmproject.com/utilities/?show=Statistics). Then, a standard MNI map of brain functional differences between DFP and HC was separately created for each study using a Gaussian kernel of 20 mm full width at half maximum (FWHM). Next, these maps were combined in a standard random-effects model considering sample size, intra-study variability and between-study heterogeneity, yielding a final map of brain functional differences (*z* map) for all included studies. Given the relatively small number of studies, we adopted a threshold of uncorrected voxel-level *P* = 0.005 and cluster extent = 20 voxels to optimally balance sensitivity and specificity^52^.

A range of supplementary analyses were pursued to test the reliability and robustness of our meta-analysis results. First, *I*^*2*^ statistic was calculated to describe which percentage of the variability between studies might be due to between-study heterogeneity, with *I*^*2*^ = 25%, 50%, and 75% indicating low, medium, and high heterogeneity, respectively^53,54^. Second, potential publication bias was assessed by funnel plots where two tests for small-study effect and excess significance were performed^55,56^. Finally, to test the repeatability of the results, we carried out a jackknife sensitivity analysis by repeating meta-analyses *K* times iteratively (*K* is the number of included studies), leaving out one study each time^57,58^. The results that remain significant in all or most of the combinations of studies are considered to be highly repeatable.

### Brain gene expression data processing

Brain gene expression data were obtained from the AHBA dataset (http://www.brainmap.org), which was derived from six human postmortem donors (Supplementary Table S1). The original expression data of more than 20,000 genes at 3702 spatially distinct brain tissue samples were processed using a newly proposed pipeline^35^. A schematic of a workflow for processing brain gene expression data is shown in Supplementary Fig. S1. Specifically, the probe-to-gene annotations were first updated based on the latest available information from the National Center for Biotechnology Information (NCBI) using the Re-Annotator package^59^. With intensity-based filtering, we excluded probes that did not exceed the background noise in at least 50% of samples across all donors. Since multiple probes were used to measure the expression level of a single gene, we further used the RNA-seq data as a reference to select probes. After excluding genes that did not overlap between RNA-seq and microarray datasets, we computed the correlations between microarray and RNA-seq expression measures for the remaining genes. After ruling out probes with low correlations (*r* < 0.2), a representative probe was selected for a gene based on its highest correlation with the RNA-seq data. Here, only the tissue samples in the left cerebral cortex were included. For one, all six donors had expression data in the left hemisphere, whereas only two donors had samples in the right hemisphere. For another, the inclusion of subcortical samples might introduce potential biases given the substantial divergence in gene expression between cortical and subcortical regions^34^. To account for potential between-sample differences and donor-specific effects in gene expression, we conducted both within-sample cross-gene and within-gene cross-sample normalization with the scaled robust sigmoid normalization method. Differential stability (DS) is a measure of consistent regional variation across donor brains. Earlier work has demonstrated that genes with high DS show more consistent spatial expression patterns between donors and are enriched for brain-related biological functions^60^. Since gene expression conservation across subjects is a prerequisite for the transcriptome-neuroimaging spatial correlations, only genes with relatively more conserved expression patterns were selected for analysis. To achieve this goal, we ranked the genes by their DS values and chose the genes with the top 50% highest DS for the main analysis. Furthermore, to evaluate the effect of different DS threshold selections, we conducted sensitivity analyses by using two other DS cutoff thresholds (top 40% and 60%). After these processing procedures, we obtained normalized expression data of 5013 genes for 1280 tissue samples. Because our neuroimaging meta-analysis was performed within a gray matter mask provided by the SDM-PSI, we further restricted our analyses to the samples within this mask, resulting in a final sample × gene matrix of 894 × 5013. The numbers of remaining probes and genes at each processing step are shown in Supplementary Fig. S2.

### Transcription-neuroimaging association analysis

To derive the DFP-related functional alteration of a given brain tissue sample, we defined a 3-mm radius sphere centered at the MNI coordinate of this sample and extracted the average *z* value of voxels within the sphere from the meta-analysis *z* map. Then, gene-wise cross-sample Pearson’s correlations between gene expression and *z* values were performed to determine genes whose expression levels were correlated with brain functional changes in DFP. Multiple comparisons were corrected using the Benjamini and Hochberg method for false discovery rate (FDR-BH) (*q* < 0.05).

To further test whether the number of the identified genes was significantly greater than the random level, a permutation test was pursued to establish the significance of our results. As transcriptional data are spatially autocorrelated, i.e., nearby anatomical regions tend to have more similar patterns of gene expression than spatially distant regions, the standard non-parametric null (i.e., randomly shuffling the sample labels) is strongly violated by the spatial autocorrelation of brain maps, yielding increased family-wise error rates^61^. To address this issue, we used a spatially constrained null model proposed by Burt et al.^62^ to conduct the permutation test. This method is implemented in an open-access, Python-based software package, BrainSMASH: Brain Surrogate Maps with Autocorrelated Spatial Heterogeneity (https://github.com/murraylab/brainsmash). It can simulate volumetric surrogate brain maps that preserve the spatial autocorrelation using three-dimensional Euclidean distance between regions. To correct the spatial autocorrelation in transcriptional data, we used this method to generate spatial autocorrelation-preserving surrogate maps for each gene. These surrogate maps were used to re-identify genes related to brain functional changes in DFP using exactly the same method as described above. Next, we repeated this procedure 5000 times and recorded the number of genes identified in each test to build a null distribution. Finally, we compared the number of genes identified using the real data with this null distribution to determine whether our results were different from random.

### Gene enrichment analysis

A series of enrichment analyses were performed for the identified genes associated with brain functional alterations in DFP. First, functional annotation was carried out with the use of the ToppGene portal (https://toppgene.cchmc.org/)^63^. Gene ontology (GO) was adopted to assess biological functions including molecular functions, cellular components and biological processes. The disease and pathway databases were used to assess the related diseases and biological pathways. Second, online tissue-specific expression analysis (TSEA) (http://genetics.wustl.edu/jdlab/tsea/) and cell type-specific expression analysis (CSEA) (http://genetics.wustl.edu/jdlab/csea-tool-2/)^64^ tools were utilized to conduct tissue, cell type, and temporal-specific expression analyses, with the aim of determining the specific tissues, cortical cell types, and developmental stages in which these genes were overrepresented. A specificity index probability (pSI) was used to index how genes are more enriched in specific terms relative to others^65^ and four pSI thresholds (0.05, 0.01, 0.001, and 0.0001) were employed in this analysis. Finally, we examined the overlap between genes associated with brain functional alterations in DFP found in the present study and schizophrenia-associated genes in the MalaCards database (https://www.malacards.org/)^66^. For the aforementioned enrichment analyses, Fisher’s exact tests were utilized to assess their significance. Multiple testing was corrected using the FDR-BH (*q* < 0.05).

### Protein-protein interaction analysis

Protein-protein interaction (PPI) analysis was performed by STRING v11.0 (https://string-db.org/) to determine whether the genes associated with brain functional alterations in DFP could construct a PPI network. All PPI pairs with a confidence interaction score > 0.9 were extracted. Genes with the top 10% highest degree values (i.e., the number of edges connected to a gene) were defined as hub genes. In addition, the Human Brain Transcriptome database (http://hbatlas.org/) was employed to characterize the spatial-temporal expression trajectory of hub genes with the highest degree values.

### Behavioral relevance analysis

To capture the behavioral relevance of the genes related to brain functional alterations in DFP, we tested the associations between gene expression and behavioral domains via the Neurosynth (https://neurosynth.org/), a well-validated and publicly available platform for meta-analysis of neuroimaging literature^67^. The Neurosynth database contains activation maps of 1335 behavioral terms that describe nearly all aspects of human behavior. For each term, cross-sample correlation analyses were performed between its activation values and gene expression measures, resulting in a set of correlation coefficients corresponding to the genes. A positive correlation coefficient indicates that a brain region with higher gene expression tends to show greater neural activation, while a negative correlation coefficient means that a brain region with lower gene expression tends to show greater neural activation. Thus, both positive and negative correlation coefficients indicate that a gene contributes to a behavioral term. To avoid biases due to offset, we averaged the absolute values of these correlation coefficients (|*r* | mean) to index the extent to which this set of genes was linked to each behavioral term. Finally, the behavioral terms were ordered based on their |*r* | _mean_ and those with the highest |*r* | _mean_ were selected to capture the behavioral relevance of the genes related to brain functional alterations in DFP. Here, a threshold of |*r* | _mean_ > 0.2 was chosen for ease of interpretability.

## Results

### Resting-state brain functional alterations in DFP

**Fig. 3:**
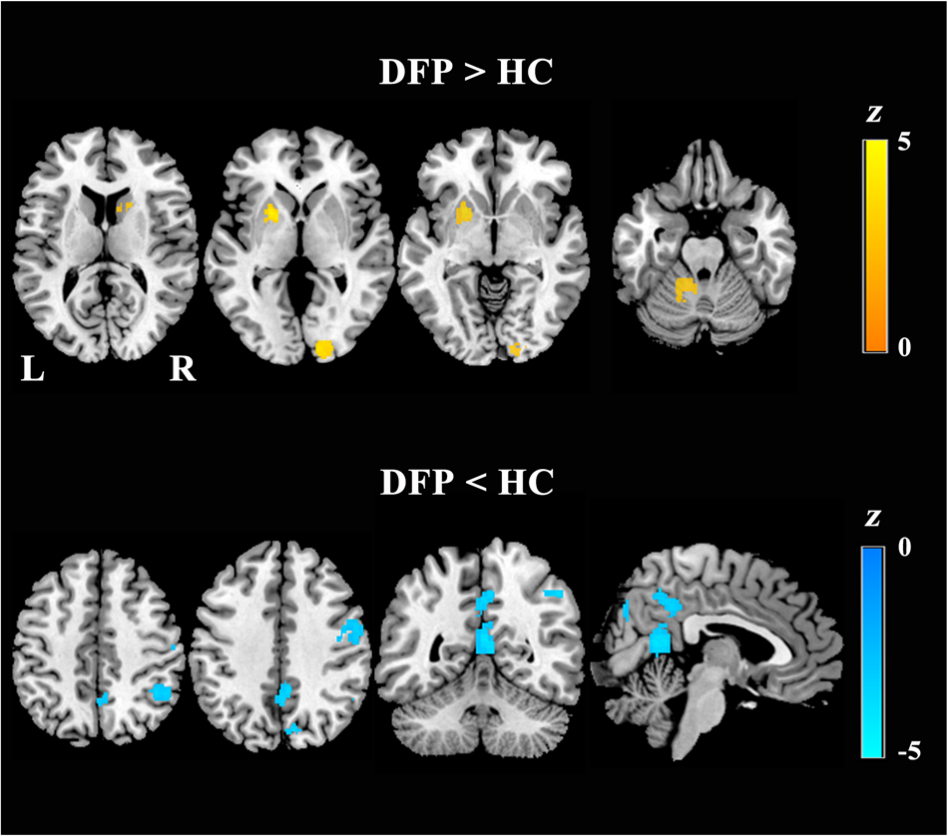
Brain regions with functional differences between DFP and HC. DFP patients with drug-naive first-episode psychosis, HC healthy controls, L left, R right.

After a comprehensive literature search and selection, our meta-analysis included 8 studies comprising 500 DFP and 469 HC. Demographic, clinical, and imaging characteristics of the included studies are shown in Table 1. Voxel-wise neuroimaging meta-analysis revealed widespread brain functional differences between DFP and HC (Fig. 3 and Table 2). In comparison with HC, DFP showed increased function in the left cerebellar hemisphere, left putamen, right calcarine sulcus, and right caudate, as well as decreased function in the bilateral posterior cingulate gyrus, bilateral precuneus, right cuneus, right precentral gyrus, and right inferior parietal lobule. Moreover, heterogeneity analysis (*I*^*2*^ < 25%) (Supplementary Table S2), publication bias analysis including funnel plots with small-study effect and excess significance tests (*P* > 0.05) (Supplementary Fig. S3 and Supplementary Table S3), and jackknife sensitivity analyses (75–100% consistency) (Supplementary Tables S4 and S5) suggested the reliability and robustness of our meta-analysis results.

**Table 1:** Demographic, clinical, and imaging characteristics of the included studies.

	Demographic characteristics				Clinical characteristics (patients only)					Imaging characteristics		
	Number/female		Age, yr				PANSS score					
Study	DFP	HC	DFP	HC	Drug status	Illness duration, mo	Total	Negative	Positive	Scanner	Software	Measures
Li et al.^19^	72/48	79/46	14.7 ± 1.78	14.3 ± 2.17	Drug naive	< 12	68.9 ± 18.4	16.3 ± 7.53	15.8 ± 5.19	3.0 T	DPABI	ALFF
Yan et al.^20^	69/19	74/29	24.22 ± 7.08	26.27 ± 6.97	Drug naive	13.74 ± 11.76	84.19 ± 8.25	17.58 ± 4.10	24.42 ± 3.88	3.0 T	DPARSF	ReHo
Zhao et al.^21^	44/13	26/9	23.7 ± 5.3	22.6 ± 3.7	Drug naive	12 ± 9.2	102 ± 16.7	24.7 ± 7.5	15.3 ± 3.3	3.0 T	DPARSF	ReHo
Wu et al.^22^	32/16	32/11	30.94 ± 8.25	31.37 ± 7.84	Drug naive	12 ± 9.2	77.38 ± 5.17	20.59 ± 3.46	20.00 ± 4.32	3.0 T	SPM8	fALFF
Liu et al.^23^	48/NA	31/NA	15.79 ± 1.64	15.42 ± 1.52	Drug naive	5.35 ± 6.12	75.10 ± 9.88	17.92 ± 6.95	21.50 ± 5.01	3.0 T	SPM8	ReHo
Lei et al.^24^	124/63	102/52	NA	NA	Drug naive	NA	NA	NA	NA	3.0 T	DPARSF	ALFF
Ren et al.^25^	100/59	100/59	24.30 ± 7.45	24.39 ± 7.58	Drug naive	6.25 ± 11.04	97.88 ± 17.75	18.84 ± 7.70	25.11 ± 5.97	3.0 T	DPARSF	ALFF
Scheef et al.^26^	11/3	25/13	32 ± 5	30 ± 6	Drug naive or free	NA	43.1 ± 8.5	21.4 ± 7.9	20.2 ± 2.9	3.0 T	SPM2	CBF

*DFP* patients with drug-naive first-episode psychosis, *HC* healthy controls, *PANSS* Positive and Negative Syndrome Scale, *NA* not available, *ALFF* amplitude of low-frequency fluctuations, *fALFF* fractional amplitude of low-frequency fluctuations, *ReHo* regional homogeneity, *CBF* cerebral blood flow.

**Table 2:** Brain regions showing functional differences between DFP and HC identified by voxel-wise meta-analysis.

Regions	Cluster size (voxels)	Peak MNI coordinate	SDM-*z*	*P* value
DFP > HC				
Left cerebellar hemisphere	187	−18, −58, −20	3.20	6.93 × 10^−4^
Left putamen	158	−20, 10, −4	4.24	1.11 × 10^−5^
Right calcarine sulcus	184	14, −94, 2	3.43	3.02 × 10^−4^
Right caudate	24	8, 8, 6	3.17	7.63 × 10^−4^
Right caudate	46	16, 14, 16	3.23	6.11 × 10^−4^
DFP < HC				
Bilateral posterior cingulate gyrus	280	4, −58, 14	−4.13	1.81 × 10^−5^
Bilateral precuneus	227	2, −46, 36	−3.97	3.56 × 10^−5^
Right cuneus	94	10, −76, 40	−3.48	2.47 × 10^−4^
Right precentral gyrus	187	54, −10, 38	−3.75	8.82 × 10^−5^
Right inferior parietal lobule	101	50, −52, 44	−3.68	1.18 × 10^−4^

*DFP* patients with drug-naive first-episode psychosis, *HC* healthy controls, *MNI* Montreal Neurological Institute, *SDM* seed-based *d* mapping.

### Genes associated with brain functional alterations in DFP

By leveraging transcription-neuroimaging spatial correlation analysis, we found that expression measures of 1662 genes were significantly linked to brain functional alterations in DFP (FDR-BH correction, *q* < 0.05) (Supplementary File 1). The spatially-constrained permutation test demonstrated that none out of 5000 permutations yielded more genes than those identified using the real data (*P* < 0.0002), indicating that our results were different from random. Moreover, we observed significant overlaps between the genes in the main analyses and those identified using two other DS cutoff thresholds of 40% (overlap ratio: 96.61%) and 60% (overlap ratio: 92.33%) (Supplementary Table S6 and Supplementary File 2).

### Gene enrichment results

**Fig. 4:**
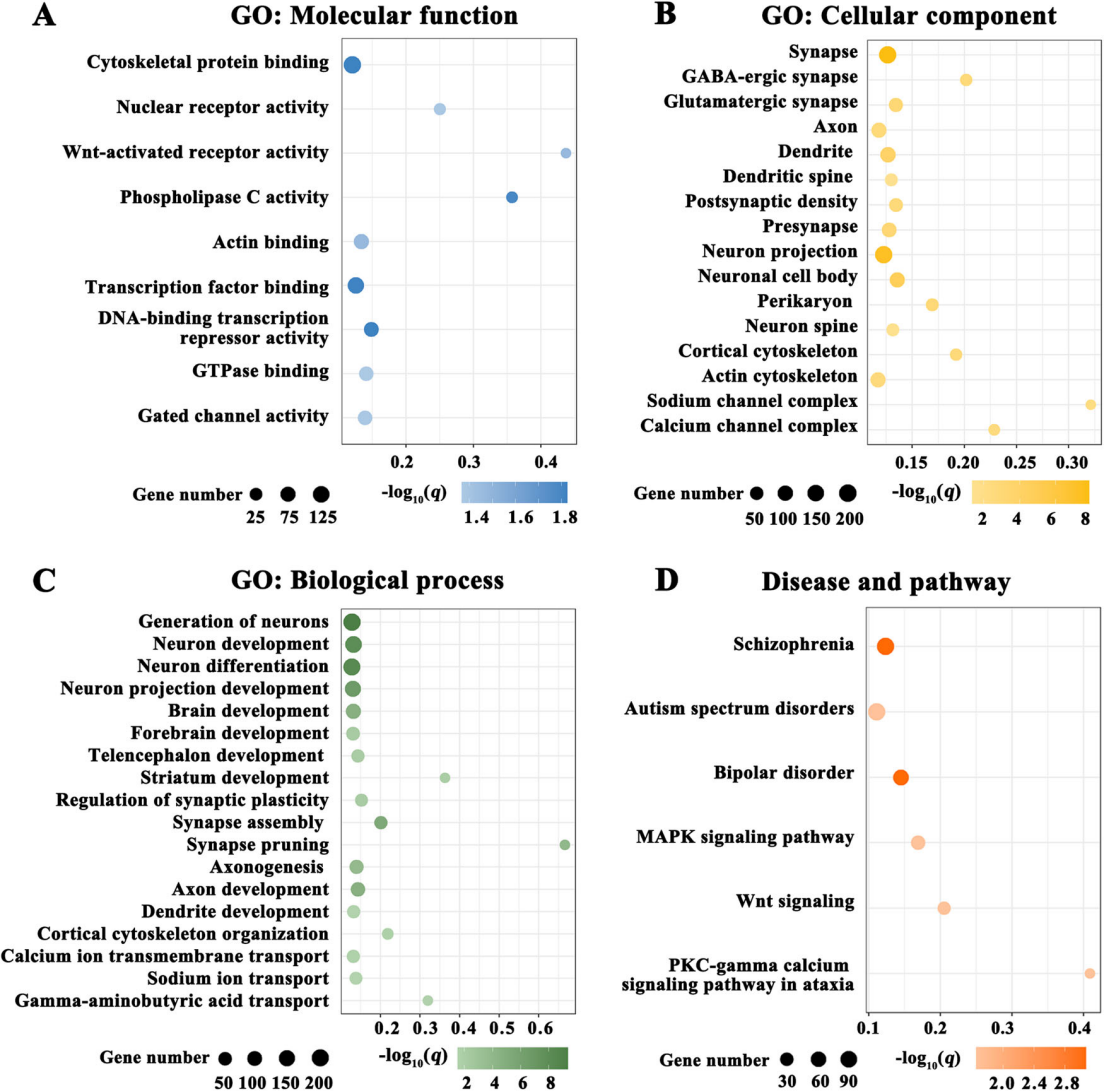
Functional enrichment of the genes associated with brain functional alterations in DFP. **A** GO: Molecular function. **B** GO: Cellular component. **C** GO: Biological process. **D** Disease and pathway. The *x* axis shows rich factor and the *y* axis shows items. The globule size represents the number of genes overlapping with those belonging to each item, and the globule color represents statistical significance. The rich factor refers to the ratio of the number of significant genes annotated to the item to the number of all genes annotated to the item. GO gene ontology, DFP patients with drug-naive first-episode psychosis.

To characterize the biological functions, diseases, and pathways of the genes associated with brain functional alterations in DFP, we performed functional enrichment analyses using the ToppGene portal. The enrichment results are listed in Supplementary File 3 and are illustrated in Fig. 4. In terms of GO, the 1662 genes were mainly enriched for molecular functions (e.g., cytoskeletal protein binding, actin binding, nuclear receptor activity, and gated channel activity) (Fig. 4A), cellular components (e.g., synapse, glutamatergic synapse, axon, dendrite, neuron, and ion channel) (Fig. 4B), and biological processes (e.g., neuron development, neuron differentiation, synapse assembly, synapse pruning, cytoskeleton organization, and ion transport) (Fig. 4C) of the cerebral cortex. With respect to diseases, the identified genes were enriched for several psychiatric disorders including schizophrenia, autism spectrum disorders, and bipolar disorder (Fig. 4D). As to pathways, these genes were enriched for MAPK signaling pathway, Wnt signaling, and PKC-gamma calcium signaling pathway in ataxia (Fig. 4D).

**Fig. 5:**
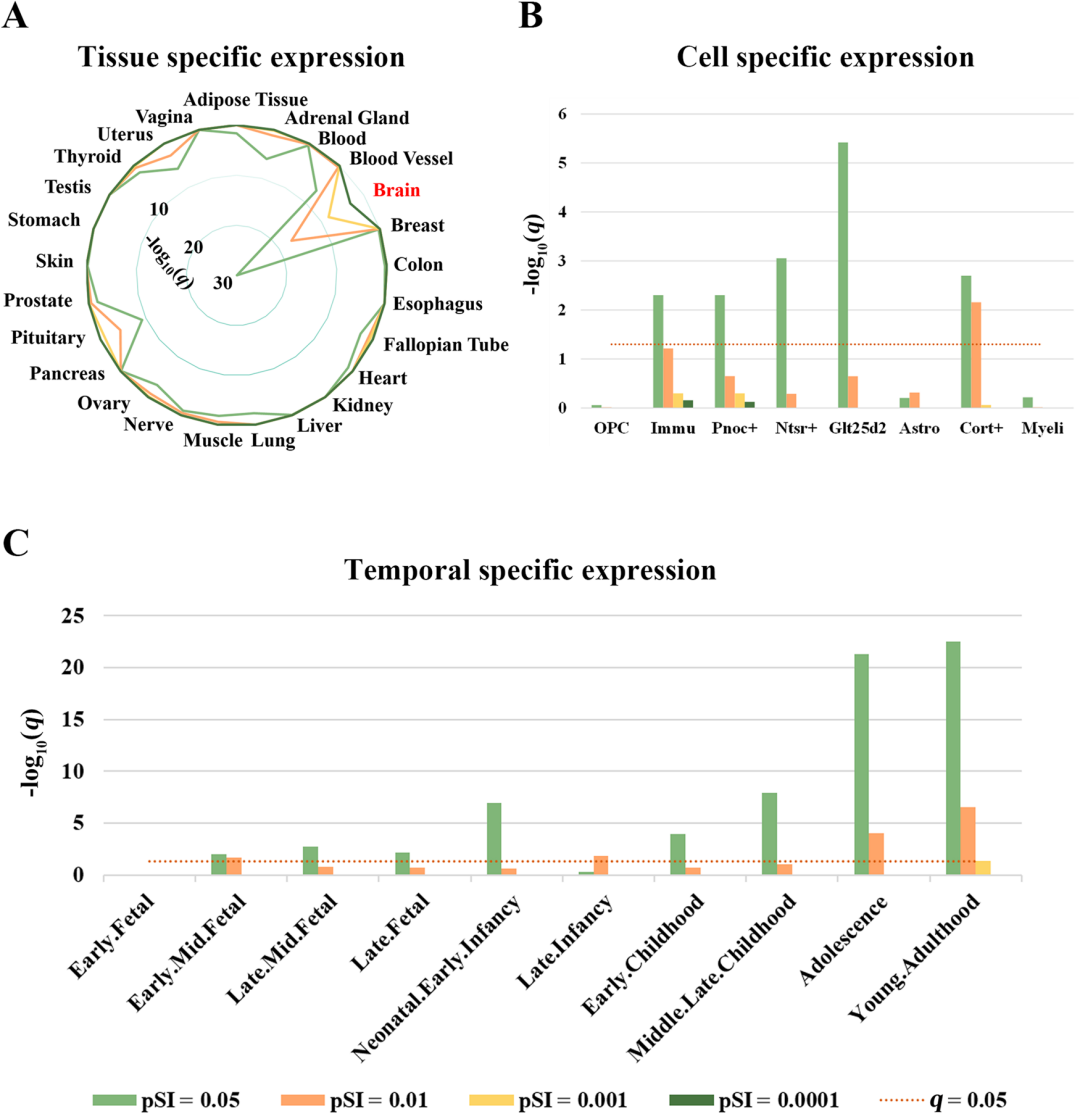
Specific expression of the genes associated with brain functional alterations in DFP. **A** Tissue-specific expression. **B** Cell-specific expression. **C** Temporal-specific expression. OPC oligodendrocyte progenitor cells, Immu immune cells, Pnoc + prepronociceptin-expressing neurons, Ntsr + corticothalamic neurons, Glt25d2 corticopontine neurons, Astro Astrocytes, Cort + corticosterone-expressing neurons, Myeli myelinating oligodendrocytes, pSI specificity index probability, DFP patients with drug-naive first-episode psychosis.

Tissue-specific expression analysis demonstrated that the 1662 genes were specifically expressed in the brain tissue (Fig. 5A and Supplementary File 4, sheet “Tissues”). Regarding cortical cell types, these genes were specifically expressed in multiple types of neurons such as Pnoc + , Ntsr + , Glt25d2, and Cort+ neurons as well as immune cells (Fig. 5B and Supplementary File 4, sheet “Cell types”). Temporal-specific expression analysis revealed that these genes were preferentially expressed during nearly all developmental periods with exception of the early fetal stage (Fig. 5C and Supplementary File 4, sheet “Developmental stages”).

Fisher’s exact test revealed that the genes related to brain functional alterations in DFP found in the present study significantly overlapped with the 400 schizophrenia-related genes from the MalaCards database (52 overlap genes, odds ratio = 1.64, *P* = 0.001), indicating some degree of psychosis specificity of our findings.

### PPI network and hub genes

**Fig. 6:**
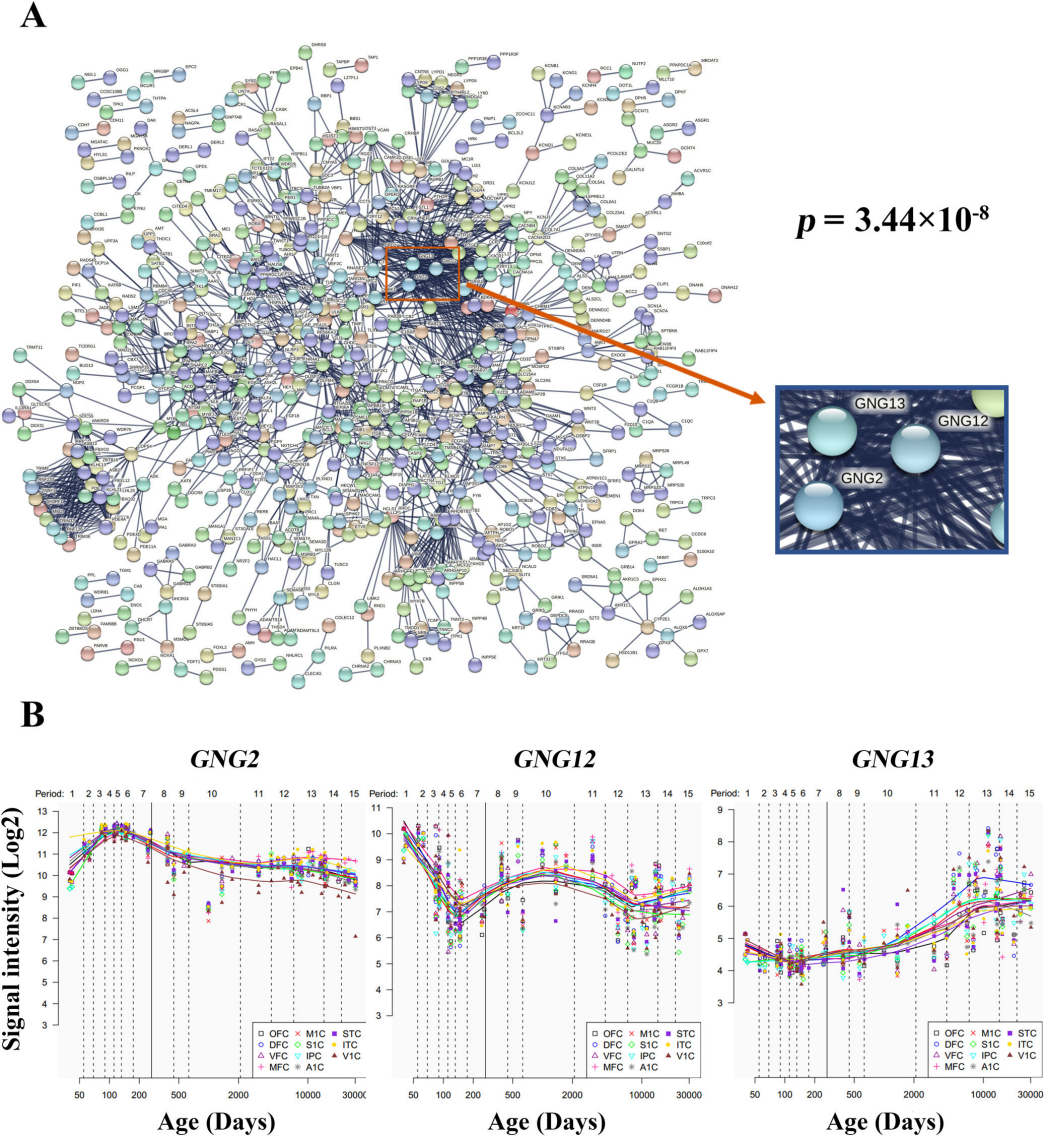
PPI network and hub genes. **A** A PPI network with 689 genes and 2151 edges. The *P* value denotes the statistical significance of how likely the proteins encoded by the input genes are connected to construct a network. **B** Spatial and temporal expression curves of three hub genes with the highest degree values (i.e., *GNG2*, *GNG12*, and *GNG13*). GNG G protein subunit gamma, PPI protein-protein interaction.

PPI analysis revealed that 689 genes from the 1662 genes could construct an interconnected PPI network (Fig. 6A). This network consisted of 2151 edges, which was significantly higher than expected (*P* = 3.44 × 10^−8^). In total, 69 genes with the top 10% highest degree values were defined as hub genes (Supplementary File 5). We also delineated the spatial-temporal expression trajectory of three hub genes with the highest degree values, i.e., G protein subunit gamma 2 (*GNG2*, degree value = 52), G protein subunit gamma 12 (*GNG12*, degree value = 51) and G protein subunit gamma 13 (*GNG13*, degree value = 49) (Fig. 6B).

### Associations with behavioral terms

**Fig. 7:**
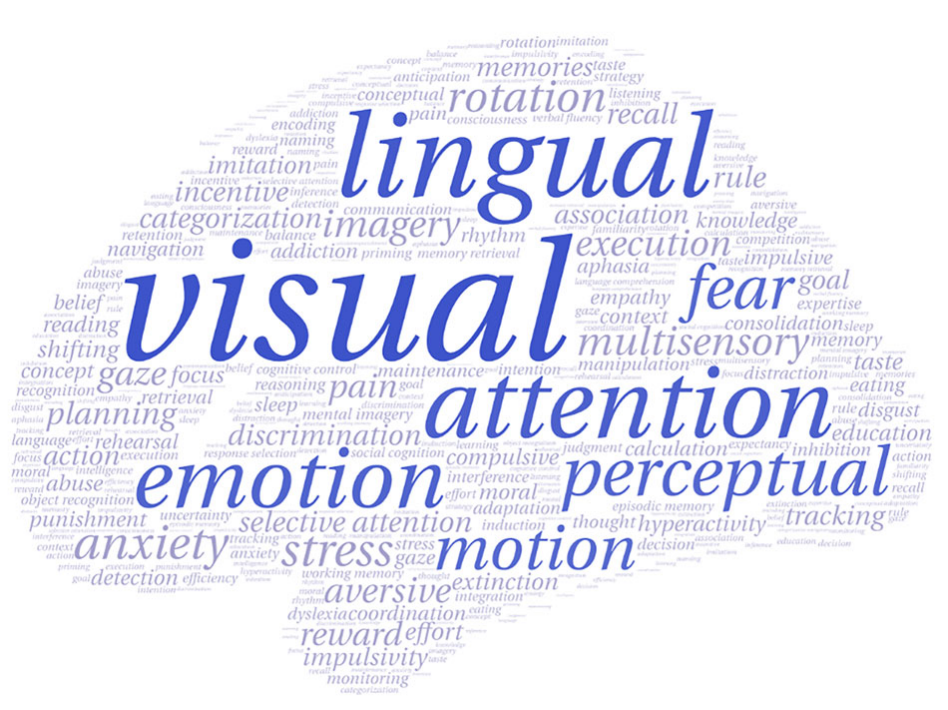
Behavioral relevance of the genes associated with brain functional alterations in DFP. Font sizes of the behavioral terms represent the correlations (|*r* | _mean_) between their activation values and gene expression measures. Blue terms are those with the highest correlations (|*r* | _mean_ > 0.2). DFP patients with drug-naive first-episode psychosis.

By linking gene expression with behavioral domains via the Neurosynth, we found that the genes associated with brain functional alterations in DFP were correlated with multiple behavioral terms including “visual”, “lingual”, “attention”, “emotion”, “perceptual”, “motion”, and “fear” (|*r* | _mean_ > 0.2) (Fig. 7 and Supplementary File 6).

## Discussion

This study opens new perspectives by being the first to investigate the genetic mechanisms underlying resting-state brain functional alterations in DFP using a combination of neuroimaging meta-analysis and transcriptome-neuroimaging spatial correlation. Meta-analysis revealed a mixture of increased and decreased resting-state brain function in widespread areas including the default-mode (posterior cingulate gyrus, precuneus, and inferior parietal lobule), visual (calcarine sulcus and cuneus), motor (precentral gyrus), striatal (putamen and caudate), and cerebellar systems in DFP. Moreover, these brain functional alterations were spatially associated with the expression of 1662 genes, which were enriched for molecular functions, cellular components, and biological processes of the cerebral cortex, as well as psychiatric disorders including schizophrenia. Specific expression analyses demonstrated that these genes were specifically expressed in the brain tissue, in cortical neurons and immune cells, and during nearly all developmental periods. Concurrently, the genes associated with brain functional alterations could construct a PPI network supported by hub genes. In addition, these genes were linked to multiple behavioral domains including emotion, attention, perception, and motor. Collectively, these findings endorse the notion that brain functional damage in psychosis involves a complex interaction of polygenes with various functional characteristics.

Our meta-analysis demonstrated that widespread brain functional abnormalities were present in DFP. Decreased brain function was observed in posterior cingulate gyrus, precuneus, and inferior parietal lobule, which serve as key components of the default-mode network (DMN). DMN is involved in the processing of various high-level cognitive functions, such as self-related cognition and memory^68,69^. In psychosis patients, DMN dysfunction may relate to overly intensive self-reference and memory impairments^70^. Hyperactivity of the striatum appears to be consistent findings across psychosis studies, thereby providing biomarkers for this disorder and targets for its treatment^71^ given that the striatum harbors the largest density of dopamine D2 receptors^72^. The calcarine sulcus and cuneus belong to the primary visual cortex, which is involved in visual processing and associated with visual hallucinations^73–75^. The precentral gyrus is a critical part of the mirror neuron system and its functional damage is believed to play an important role in social cognition impairment in schizophrenia^76,77^. Our observation of increased function in the cerebellum supports the concept that disrupted synchronization in the cerebellar prefrontal circuitry underlies negative symptoms and possibly also cognitive dysfunction in psychosis^78^. Notably, since our meta-analysis focused on DFP and was not contaminated by confounding factors such as medication use and/or long illness duration, the present findings may provide a more refined picture of resting-state brain functional alterations specific to psychosis.

Further transcriptome-neuroimaging spatial correlation analyses revealed that the brain functional alterations in DFP were related to expression of 1662 genes, which were enriched for molecular functions, cellular components, and biological processes of the cerebral cortex. Indeed, damaging mutations in genes that are critical to synaptic structure, function, and plasticity play a prominent role in the pathophysiology of schizophrenia^79^. For instance, disruptions in the synaptic structural organization are critical to the development of psychosis. The postsynaptic density is a vital component of dendritic spines, with marked thickening in postsynaptic membrane of excitatory synapses and a lack in the symmetric inhibitory synapses^80,81^. The postsynaptic density has been strongly implicated in psychosis at both genetic and protein levels^82–85^. In addition, remodeling of dendritic trees and dendritic spines has been observed as a result of environmental and genetic influences acting both early and later in life in schizophrenia^86^. Alterations in dendritic morphology, such as reduction in the size of dendritic arborization, have been described in postmortem samples from patients with schizophrenia^87^. There is ample evidence that DFP is associated with elevations in glutamatergic metabolites across several brain regions, which may reflect increases in glutamatergic synaptic activity^88^. Several more recent magnetic resonance spectroscopy studies have documented glutamate alterations in the salience network^89^ and hippocampus^90^ in DFP. These previous findings are consistent with the current observation of significant gene enrichment for glutamatergic synapse. The mature neuron is composed of multiple subdomains, each with a distinctive cytoskeletal organization^91^. Actinin plays an important role in the regulation of cytoskeletal remodeling and the inactivation of the N-methyl-D-aspartate receptor (NMDAR) for glutamate, which have been implicated in psychosis by influencing brain development and plasticity processes^92,93^. Moreover, reelin is found to stabilize the actin cytoskeleton by inducing cofilin phosphorylation; decreased reelin expression in the mature brain causes destabilization of neurons and their processes, leading to aberrant plasticity and aberrant wiring of brain circuitry, which may be related to psychosis^94^. Ion channels are vital for neuronal functions, triggering nerve impulses, and neurotransmitter release^95^. Voltage-gated sodium channels are broadly distributed in the central nervous system and are linked to neuronal excitability, with their dysfunction being implicated in psychosis pathogenesis^96,97^. Furthermore, calcium signaling has been noted to be of particular relevance in the etiology of schizophrenia^98–100^. GWAS have indicated that genetic variations associated with the calcium signaling pathway can increase the risk of developing schizophrenia^101^. Aside from a better understanding of the molecular basis of brain functional alterations in psychosis, these findings might hold clinical value and translational potential. It is largely known that all drugs currently licensed to treat psychosis are dopamine D2/3 blockers^102^. Although helpful for reducing psychotic symptoms, these agents are not curative and generally do not address the neurocognitive and social difficulties inherent to the disorder^103^. Our data indicate that novel interventions designed to remediate disruptions in synaptic structural organization potentially offer more specific therapeutic benefits.

These genes were enriched for psychiatric disorders including schizophrenia, autism spectrum disorders, and bipolar disorder, in favor of the idea that these conditions with several common clinical features may share some similar genetic basis. The enriched pathways include MAPK signaling pathway, Wnt signaling and PKC-gamma calcium signaling pathway in ataxia. Previous studies have documented that MAPK signaling alterations could result from a protein dephosphorylation defect in vivo and might be involved in the pathology of psychosis^104,105^. Zhang et al. reported that PKC γ-mediated phosphorylation signal was impaired in Hint1-deficient neuron, which might contribute to the pathogenesis of psychosis^106^. Furthermore, there is empirical evidence for the crucial role of Wnt signaling pathway in neurodevelopment and in regulating the function and structure of the adult nervous system. Prior research shows that psychosis is characterized by abnormal Wnt gene expression and plasma protein levels, suggesting that drugs targeting the Wnt pathway may have a role in the treatment of this disorder^107^.

Specific expression analysis demonstrated that the genes associated with brain functional alterations in DFP were specifically expressed in the brain tissue and neurons, which may enhance our confidence in interpreting our findings. Besides, these genes showed specific expression in immune cells, in accordance with the previous finding that cognitive deficits in psychosis may be associated with altered expression of molecules that regulate immune cell trafficking^108^. Critically, these genes were found to be specifically expressed during nearly all developmental periods, which is coherent with the “2-hit” hypothesis of schizophrenia^109^. In that model, genetic or environmental factors disrupt early brain development (i.e., “first hit” during pre- and perinatal periods). These early disruptions produce long-term vulnerability to a “second hit” during the periods of adolescence and young adulthood, which may ultimately give rise to full-blown psychosis^110–112^.

The genes associated with brain functional alterations could construct a PPI network supported by hub genes, e.g., G protein-related genes. Many neurotransmitters involved in schizophrenia act through metabotropic G protein-coupled receptors (GPCRs). Moreover, the GPCRs for serotonin, dopamine, and glutamate have been traditionally recognized as molecular targets for antipsychotics^103^. These receptors mediate slow synaptic transmission by modulating intracellular signal transduction and induction of gene expression to exert antipsychotic action^113,114^. By correlating gene expression with behavioral terms via the Neurosynth, we found that the genes associated with brain functional alterations in DFP were linked to multiple behavioral terms including emotion, attention, perception, and motor. Echoing this finding, extensive literature has provided strong evidence that psychosis patients present with deficits in these behavioral domains^115–122^.

This study has several limitations that should be considered. First, the meta-analysis *z* map cannot represent the exact nature of resting-state brain functional differences between DFP and HC across the whole brain, as only peak coordinates and corresponding effect sizes of significant clusters in the previous studies were utilized. Our preliminary results warrant further validation by conducting analysis in a large cohort of DFP. Second, a relatively liberal statistical threshold (uncorrected voxel-level *P* = 0.005 and cluster extent = 20 voxels) was set for neuroimaging meta-analysis, considering the small number of included studies and the more recent work demonstrating that small *P* values may not yield robust findings^123^. Third, the gene expression data were derived from the AHBA postmortem brains, while the neuroimaging data were obtained from living brains of DFP. This concern may be mitigated by focusing on genes with more conservative expression patterns, but this may obscure correlations with genes exhibiting variable expression patterns across individuals. Finally, given limited gene expression data in the right hemisphere and considerable differences in gene expression between cortical and subcortical regions, our study only considered the tissue samples in the left cerebral cortex, which may introduce potential biases.

In summary, our neuroimaging meta-analysis demonstrated that DFP manifested a mix of increased and decreased resting-state brain function in widespread areas including the default mode, visual, motor, striatal, and cerebellar systems. Combined with the brain-wide transcriptome, we found that these brain functional alterations were spatially associated with the expression of 1662 genes, which showed a rich range of functional characteristics. These findings may shed light on the genetic mechanisms underlying resting-state brain functional alterations in psychosis.

## Supplementary information

Supplementary materials

Supplementary file 1

Supplementary file 2

Supplementary file 3

Supplementary file 4

Supplementary file 5

Supplementary file 6

## Data Availability

The Allen Human Brain Atlas is available at https://human.brain-map.org/static/download. The MalaCards database is available at https://www.malacards.org/. The Neurosynth database is available at https://neurosynth.org/. All other data supporting the findings of this study are available from the corresponding author on reasonable request.

## Code availability

All relevant analysis scripts are available from the corresponding author upon request.
