# Supplementary material for: Resting-state brain functional alterations and their genetic mechanisms in drug-naive first-episode psychosis
Source: Schizophrenia (Heidelb). 2023 Feb 25;9(1):13. doi: 10.1038/s41537-023-00338-z (PMC9968350; doi:10.1038/s41537-023-00338-z)
Supplement: Supplementary file 1 — Supplementary materials [file 41537_2023_338_MOESM1_ESM.doc]

**Supplementary materials**

Title: **Resting-state brain functional alterations and their genetic mechanisms in drug-naive first-episode psychosis**

**Supplementary tables and figures**

**Table S1.** Demographic information of the six adult donors in the AHBA

| **Donor** | **Age (years)** | **Gender** | **Ethnicity** | **Hemisphere** | **Post-mortem interval (h)** |
| --- | --- | --- | --- | --- | --- |
| H0351.2001 | 24 | Male | African American | Both | 23 |
| H0351.2002 | 39 | Male | African American | Both | 10 |
| H0351.1009 | 57 | Male | Caucasian | Left | 25.5 |
| H0351.1012 | 31 | Male | Caucasian | Left | 17.5 |
| H0351.1015 | 49 | Female | Hispanic | Left | 30 |
| H0351.1016 | 55 | Male | Caucasian | Left | 18 |

Abbreviation: AHBA, Allen Human Brain Atlas.

**Table S2.** The *I^2^* statistics of all reported brain regions in meta-analysis

|  | **Brain regions** | ***I*^2^** |
| --- | --- | --- |
| **DFP > HC** | Left cerebellar hemisphere | 12.92% |
|  | Left putamen | 9.48% |
|  | Right calcarine sulcus | 13.88% |
|  | Right caudate (24 voxels) | 21.58% |
|  | Right caudate (46 voxels) | 15.64% |
| **DFP < HC** | Bilateral posterior cingulate gyrus | 0.99% |
|  | Bilateral precuneus | 3.11% |
|  | Right cuneus | 2.97% |
|  | Right precentral gyrus | 6.78% |
|  | Right inferior parietal lobule | 2.92% |

Abbreviations: DFP, patients with drug-naive first-episode psychosis; HC, healthy controls

**Table S3.** The tests for small-study effect and excess significance

|  | **Brain regions** | ***p* (small-study effect test)** | ***p* (excess significance test)** |
| --- | --- | --- | --- |
| **DFP > HC** | Left cerebellar hemisphere | 0.794 | 0.849 |
|  | Left putamen | 0.554 | 0.508 |
|  | Right calcarine sulcus | 0.271 | 0.548 |
|  | Right caudate (24 voxels) | 0.658 | 0.837 |
|  | Right caudate (46 voxels) | 0.677 | 0.722 |
| **DFP < HC** | Bilateral posterior cingulate gyrus | 0.599 | 0.281 |
|  | Bilateral precuneus | 0.937 | 0.938 |
|  | Right cuneus | 0.808 | 0.728 |
|  | Right precentral gyrus | 0.139 | 0.711 |
|  | Right inferior parietal lobule | 0.743 | 0.840 |

Abbreviations: DFP, patients with drug-naive first-episode psychosis; HC, healthy controls.

**Table S4.** Jackknife sensitivity analyses of the brain regions with increased function in DFP

| **DFP > HC** | | | | | |
| --- | --- | --- | --- | --- | --- |
|  | **Left cerebellar hemisphere** | **Left putamen** | **Right calcarine sulcus** | **Right caudate (24 voxels)** | **Right caudate (46 voxels)** |
| Li et al., 2020 | 0 | 1 | 1 | 0 | 0 |
| Yan et al., 2020 | 1 | 1 | 1 | 1 | 1 |
| Zhao et al., 2019 | 1 | 1 | 1 | 1 | 1 |
| Wu et al., 2019 | 0 | 1 | 1 | 0 | 0 |
| Liu et al., 2018 | 1 | 1 | 1 | 1 | 1 |
| Lei et al., 2015 | 1 | 1 | 1 | 1 | 1 |
| Ren et al., 2013 | 1 | 1 | 0 | 1 | 1 |
| Scheef et al., 2010 | 1 | 1 | 0 | 1 | 1 |
| Total | 6 | 8 | 6 | 6 | 6 |

1 and 0 represent presence and absence of the brain region after leaving out the corresponding study. Abbreviations: DFP, patients with drug-naive first-episode psychosis; HC, healthy controls.

**Table S5.** Jackknife sensitivity analyses of the brain regions with decreased function in DFP

| **DFP < HC** | | | | | |
| --- | --- | --- | --- | --- | --- |
|  | **Bilateral posterior cingulate gyrus** | **Bilateral precuneus** | **Right cuneus** | **Right precentral gyrus** | **Right inferior parietal lobule** |
| Li et al., 2020 | 1 | 1 | 0 | 1 | 1 |
| Yan et al., 2020 | 1 | 1 | 0 | 1 | 1 |
| Zhao et al., 2019 | 1 | 1 | 1 | 1 | 1 |
| Wu et al, 2019 | 1 | 1 | 1 | 1 | 1 |
| Liu et al., 2018 | 1 | 1 | 1 | 0 | 1 |
| Lei et al., 2015 | 1 | 1 | 1 | 1 | 0 |
| Ren et al., 2013 | 0 | 1 | 1 | 1 | 0 |
| Scheef et al., 2010 | 1 | 1 | 1 | 1 | 1 |
| Total | 7 | 8 | 6 | 7 | 6 |

1 and 0 represent presence and absence of the brain region after leaving out the corresponding study. Abbreviations: DFP, patients with drug-naive first-episode psychosis; HC, healthy controls.

**Table S6.** Numbers of the genes related to brain functional alterations in DFP identified using two other DS cutoff thresholds and overlap with those in the main analyses

| **Sensitivity analyses** | **Gene number** | **Overlap genes** | **Overlap ratio** |
| --- | --- | --- | --- |
| DS threshold of 40% | 1534 | 1482 | 96.61% |
| DS threshold of 60% | 1734 | 1601 | 92.33% |

Abbreviations: DFP, patients with drug-naive first-episode psychosis; DS, differential stability.


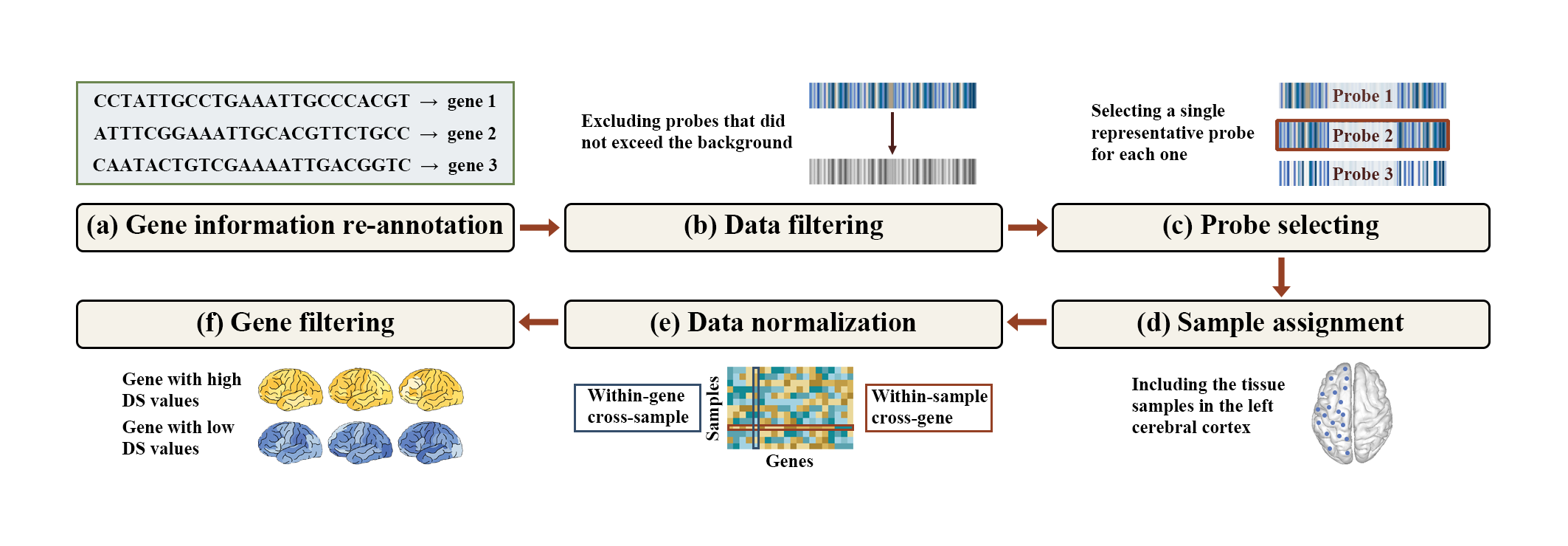


**Fig. S1** **Schematic of a workflow for processing brain gene expression data.** (a) Probe-to-gene annotations were updated based on the latest available information. (b) Probes that did not exceed the background noise in at least 50% of samples across all donors were excluded. (c) For genes indexed by multiple probes, a representative probe for a gene was selected based on the highest correlation to the RNA-seq data. (d) Sample assignment was performed and only tissue samples in the left cerebral cortex were included. (e) Both within-sample cross-gene and within-gene cross-sample normalizations were performed. (f) The 50% of the highest DS genes were chosen. Abbreviations: DS, differential stability.


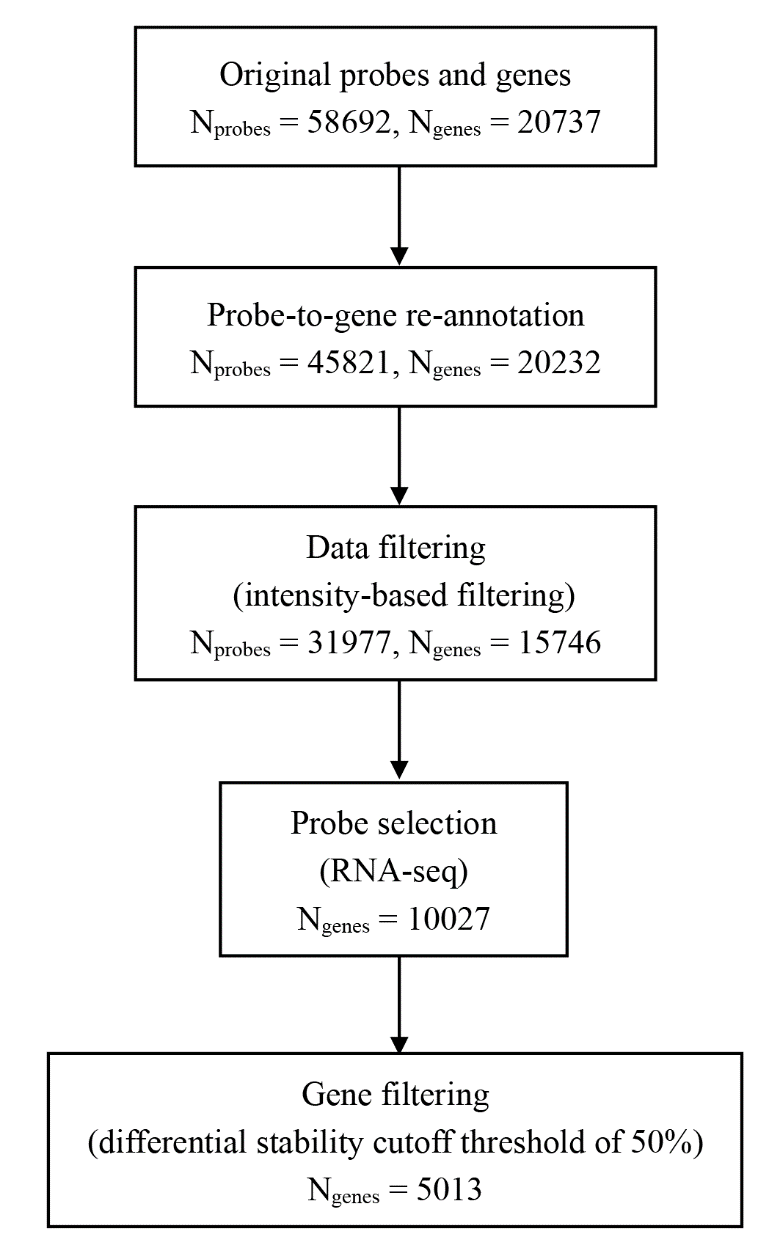


**Fig. S2 The remaining probes and genes at each processing step.**

**
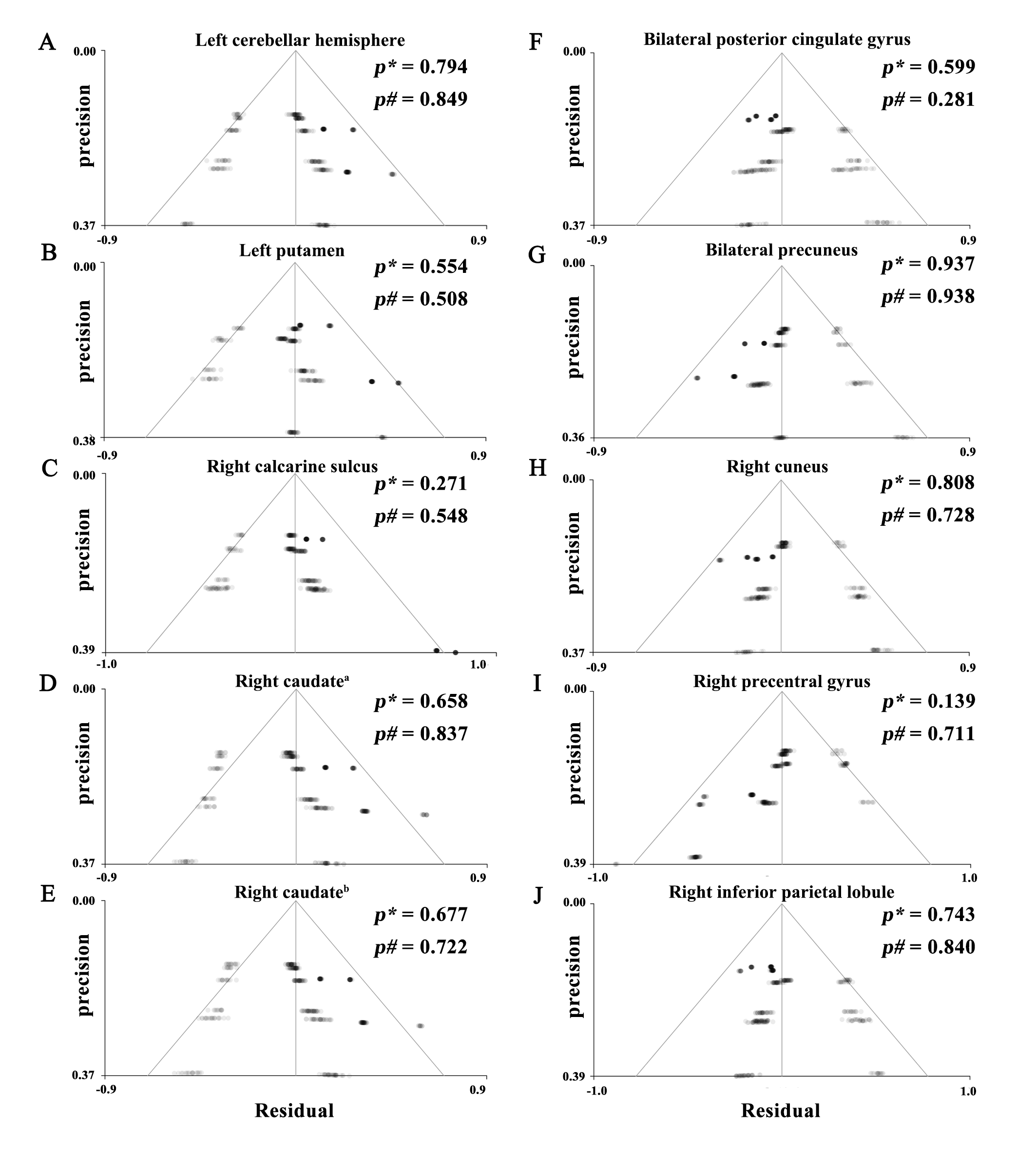
**

**Fig. S3 Funnel plots of the significant clusters showing differences in resting-state brain function between DFP and HC.** Abbreviations: DFP, patients with drug-naive first-episode psychosis; HC, healthy controls.

^*^ A test for small-study effect; ^#^ A test for excess significance.

^a^ Cluster size is 24 voxels; ^b^ Cluster size is 46 voxels.
